# Supplementary material for: BLBP Is Both a Marker for Poor Prognosis and a Potential Therapeutic Target in Paediatric Ependymoma
Source: Cancers (Basel). 2021 Apr 27;13(9):2100. doi: 10.3390/cancers13092100 (PMC8123630; doi:10.3390/cancers13092100)
Supplement: Supplementary file 1 [file cancers-13-02100-s001.zip › cancers-1182803-supplementary.pdf]

Supplementary figures

a

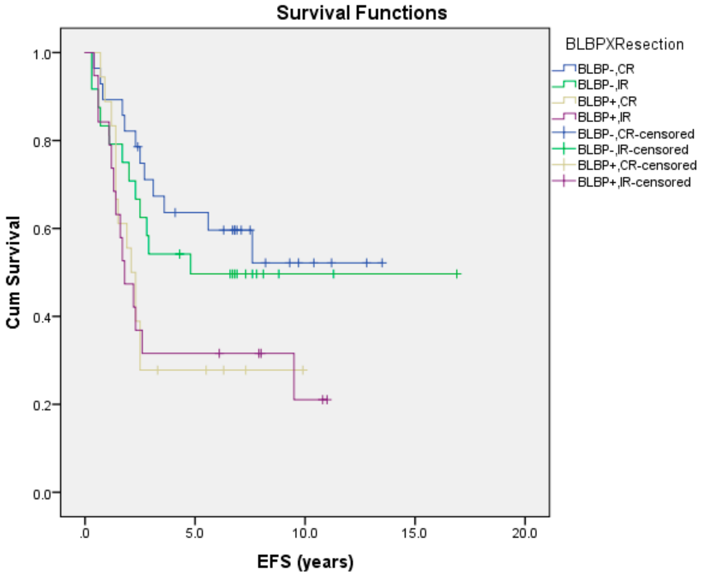

b

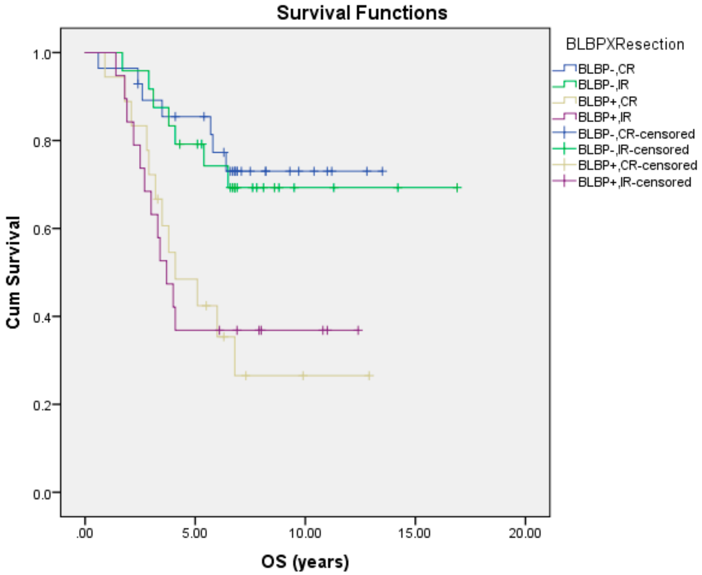

Overall Comparisons

	Chi-Square	df	Sig.
Log Rank (Mantel-Cox)	8.463	3	.037

Test of equality of survival distributions for the different levels of BLBPXResection.

Overall Comparisons

	Chi-Square	df	Sig.
Log Rank (Mantel-Cox)	15.075	3	.002

Test of equality of survival distributions for the different levels of BLBPXResection.

Figure S1: BLBP is a significant prognostic marker irrespective of the extent of resection in **a** the chemotherapy-led CNS9204 trial and **b** the radiotherapy-led CNS9904

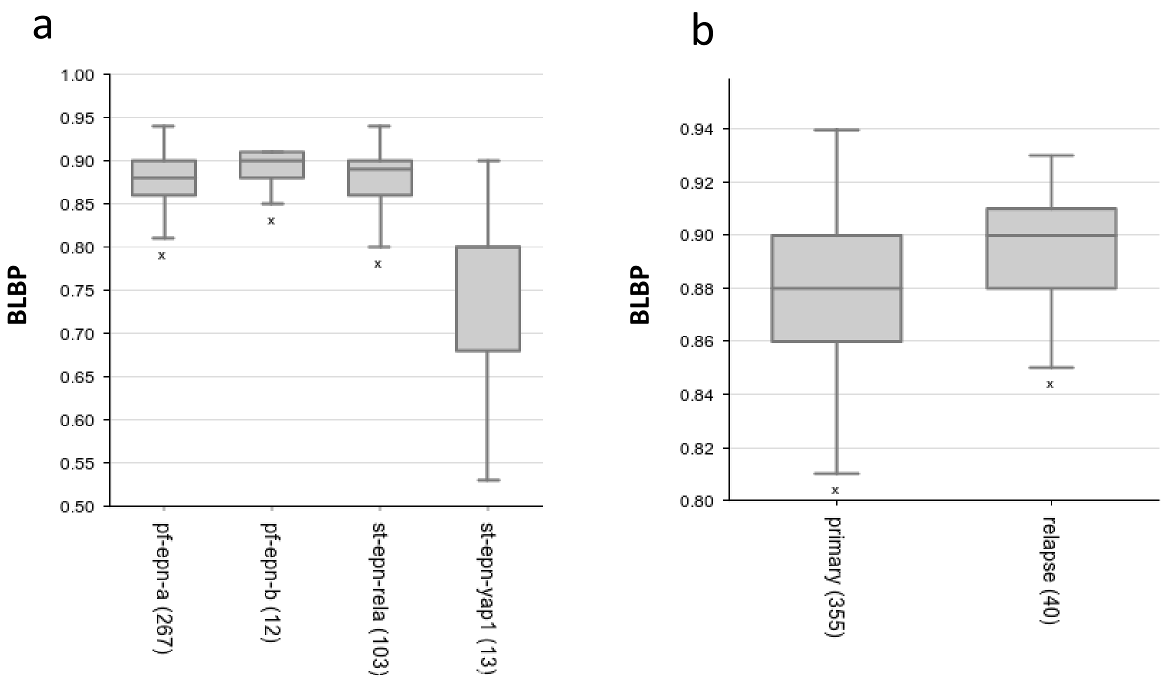

Figure S2: *BLBP* gene expression across paediatric ependymoma molecular subgroups
BLBP gene expression was analysed across the different molecular subgroups using the Kool data set (Tumor Ependymoma - Kool - 562 - custom - ilmnhm450) at R2: Genomics Analysis and Visualization Platform (http://r2.amc.nl http://r2platform.com **a** BLBP is expressed across all 4 paediatric ependymoma molecular subgroups, with only the ST-EPN-YAP1 subgroup showing significantly lower expression (ANOVA p=2e27). **b** BLBP expression is significantly increased in relapsed relative to primary samples from these subgroups (ANOVA p=0.012).

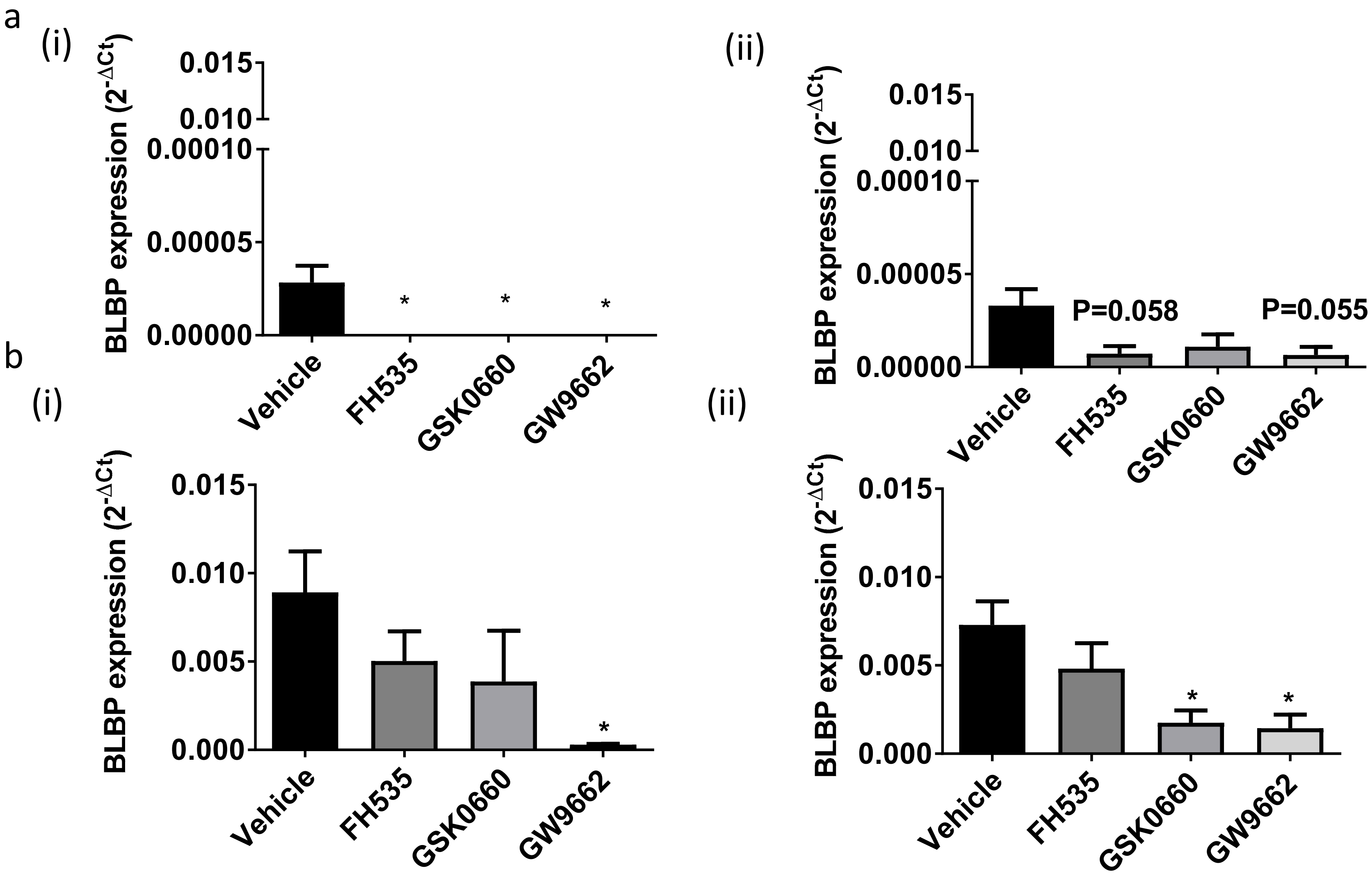

Figure S3: PPAR antagonists inhibited BLBP gene expression in ependymoma cell lines Both the ependymoma cell lines were treated with the 3 PPAR antagonists; the dual PPAR-(γ/δ) antagonist FH535, the PPAR-δ antagonist GSK0660 and the PPAR- γ antagonist GW9662. The effect on BLBP expression was measured by QRT-PCR analysis **a.** In the $BLBP^{lo}$ BXD-1425EPN cell line, BLBP expression levels significantly diminished at 48 **(i);** however, an increase was recorded at 72 hours **(ii)**. **b.** In the $BLBP^{hi}$ DKFZ-EP1 cell line, the PPAR-γ antagonist GW9662 significantly inhibited BLBP expression at both 96hrs **(i)** and 144hrs **(ii)**. The inhibitory effects of both the PPAR-δ antagonist GSK0660 and dual PPAR-(γ/δ) antagonist FH535 were found to be variable. (n=3)

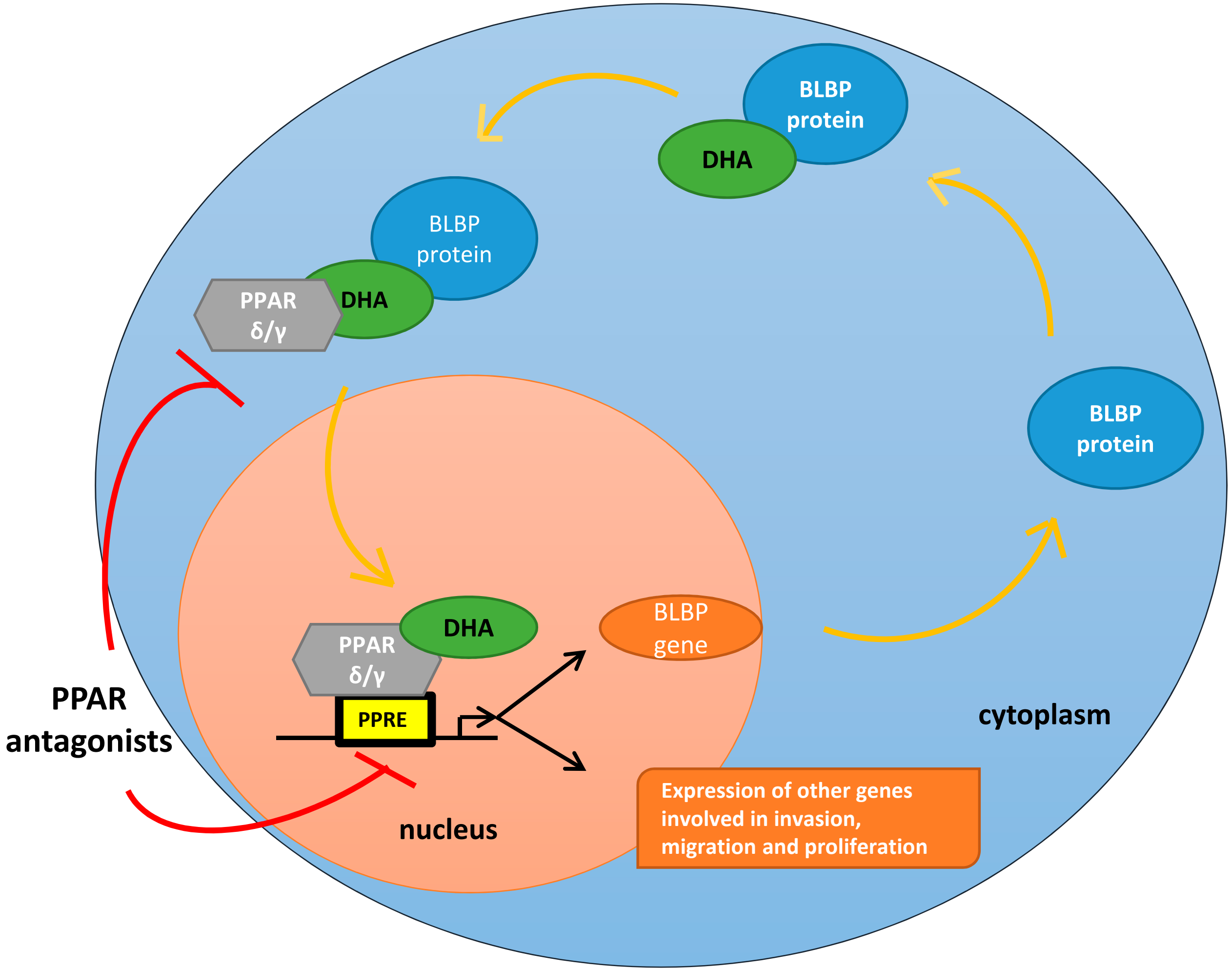

Figure S4: PPAR antagonists inhibit transcription and function of BLBP PPARs (γ and δ subtype) are transcription factors, which are essential for regulating the transcription as well as function of BLBP. In cancer cells, the nuclear localization of the fatty acid chaperoned by BLBP is essential for transcription of genes by PPARs involved in proliferation and migration of cells. Employing PPAR antagonists can therefore block both the expression of BLBP as well as other genes transcribed by PPARs.
